# Organizational Change in Complex Systems: Organizational and Leadership Factors in the Introduction of Open Dialogue to Mental Health Care Services

**DOI:** 10.1007/s10597-022-00984-0

**Published:** 2022-05-18

**Authors:** Elizabeth Lennon, Liza Hopkins, Rochelle Einboden, Andrea McCloughen, Lisa Dawson, Niels Buus

**Affiliations:** 1 The Leader Factor Pty Ltd., Sydney, NSW Australia; 2 Alfred Health, Bentleigh, Victoria Australia; 3 Faculty of Medicine and Health, The University of Sydney Susan Wakil School of Nursing and Midwifery, Sydney, NSW Australia; 4 Sydney Children’s Hospital Network, Sydney, NSW Australia; 5 School of Nursing and Midwifery, Faculty of Medicine, Nursing and Health, Monash University, Clayton, Victoria Australia; 6 Institute of Regional Health Research, University of Southern Denmark, Odense, Denmark; 7 Monash Nursing and Midwifery, Monash University, Clayton Campus, Level 1, 10 Chancellors Walk, Wellington Road, 3800 Clayton, VIC Australia

**Keywords:** Adaptive leadership, Complex systems, Open dialogue, Organizational change, Public mental healthcare services

## Abstract

Conventional mental health services are frequently criticized for failing to support people and communities in their care. Open Dialogue is a non-conventional humanistic approach to mental health care, which has been implemented in many different settings globally. At two Australian public health care services, implementation of the approach led to positive client outcomes and sustained organizational and clinical change. The aim of the study was to identify and explore the organizational, management, leadership and cultural factors that contributed to sustained implementation in these complex systems. We conducted nine individual semi-structured interviews of health care leaders and managers from the two sites. Transcriptions of the interviews were analyzed thematically. Leaders facilitated a gradual development of clinical and organizational legitimacy for the non-standardized Open Dialogue approach by holding the anxiety and frustration of practitioners and parts of the administration, cultivating cultural change and adaptation and by continually removing organizational obstacles.

## Introduction

Public mental health services are facing increasing challenges in meeting ever-rising demand for service within limited resource environments. There is a pressing need for innovation and improvement in mental health services that are sometimes described as “broken” (State of Victoria, 2021) and for greater person-centeredness and family involvement in care design and delivery. Bureaucratically and culturally generated internal and external constraints, however, often hamper efforts to implement significant and sustained change, resulting in change efforts, which fall short of service system overhaul. While the evidence-base for service effectiveness continues to grow, translational efforts to implement new approaches lag behind. In this paper, we argue that standard models of organizational change and leadership action are insufficient to enact the complex systemic change required to implement humanistic person-centered care in mental health. Using a case study approach, we examine the organizational change processes involved in implementing an alternative approach to mental health care, Open Dialogue, within conventional mental health care services.

Unlike most conventional deficit-oriented psychiatric treatment, Open Dialogue is a resource-oriented model (Priebe et al., 2014) for mental health care. It aims to mobilize psychosocial resources (coping skills, social supports, mastery of stressful events) in the social network (e.g. family, friends) of a person experiencing some form of psychosocial crisis (impacting psychological and social behaviors). Open Dialogue practitioners neither reject nor give privilege to biological views of mental illness. They respond to the client as part of a relational social system with capacity to take part in decisions about their life and therapy. One important element of its design is the flattening of therapeutic hierarchies and the inclusion of paid, peer workers is often regarded as a vital aspect of the approach (Bellingham et al., 2018) and reinforces the collaborative mindset embedded in Open Dialogue and its respect for the lived experience of clients. Being a radically person-centered approach, Open Dialogue has been read as well-aligned with UN’s human rights, which are gaining increasing importance as a global standard for mental health care (von Peter et al., 2019). The collaborative approach includes a particular dialogical psychotherapy as well as an emphasis on organizing responsive and seamless healthcare pathways (Seikkula & Arnkil, 2006). By its very nature Open Dialogue disrupts the traditional medical models of care and supporting administrative and clinical systems.

The original development of Open Dialogue in Western Lapland included a gradual, but substantial re-organization of psychiatric services, which led to changes in the structure, organization and style of management of these services to accommodate Open Dialogue practices (Haarakangas et al., 2007). Research in Open Dialogue approaches is promising and suggests significant improvement in client outcomes as well as improvement in client and family experience of care (Bergström et al., 2018; Buus et al., 2019; Buus & McCloughen, 2021).

For several decades, Open Dialogue approaches have been implemented in health care and social care sites across the world (Buus et al., 2017, 2021). Scandinavian implementation studies indicate that adoption of Open Dialogue – like any other organizational change – can generate organizational, professional, and personal resistance which may compromise the core Open Dialogue principles and methods offered at a given site (Brottveit, 2013; Søndergaard, 2009). An Australian study of implementation of Open Dialogue in a private healthcare setting observed tension at the boundaries of organizational systems, challenges between core clinical values, and conflicting expectations of professional practice and performance (Dawson et al., 2019). A recent ‘scoping review’ (Arksey & O’Malley, 2005) of Open Dialogue implementation studies (Buus et al., 2021) found that published studies did not include rich descriptions of the organizational implementation contexts (for instance culture, resourcing and management/leadership factors) or the organizational strategies for the implementation, which made it difficult to draw conclusions regarding what types of implementation would be more effective than others, and under what circumstances. Further, the review highlighted the implementation challenges linked to the flexible and needs-adapted character of the approach that gives it a spirit of indeterminacy (Buus et al., 2021). Open Dialogue is not a manualized treatment method, which can generate tensions in organizational implementation contexts that generally favor specific and standardized practices, such as fidelity measures and manualized protocols for practice (Waters et al., 2021).

Given the central role the client plays in treatment planning in this approach, Open Dialogue requires an openness to the insights of different practitioners and the client’s network, as well as high levels of collaboration with clients, and their formal and informal support systems, and a tolerance for uncertainty in clinical decision making processes. In contrast, at the center of conventional mental health services is the relatively exclusive relationship between ‘the patient’ and the medical professional, mediated by diagnosis and a treatment regime derived from Western medical practices (Foucault, 1997). This structure and organizational approach to health service management is consistent with a professional bureaucracy (Mintzberg, 1979). The structure and the associated management and leadership style emphasize expertise and specialist driven organizational design, embedded in an hierarchical administrative structure with clear lines of authority, decision-making and delegation. The focus is on resource and risk management, standards of practice, and the autonomy and decision making discretion of the expert, mediated by their technical skills, and shared knowledge and values of evidence-based medicine.

While this conventional model delivers expertise, high standards, professional autonomy and significant decisional discretion at the frontline, particularly for medical practitioners, there are also significant limitations. The structure and management culture drives a system that privileges individual care and centralized decision-making over collaboration and a genuinely multi-disciplinary, inclusive approach to knowledge sharing and treatment. The professional bureaucracy is also vulnerable systemically through its limited ability to respond to changed contexts and uncertainty through innovation and adaptation (Mintzberg, 1979). A cultural lens to understanding organizations and organizational change (Schein, 2015; Van Buskirk & McGrath, 1999), as well as systemic and psychodynamic perspectives (Armstrong, 2005; Neumann & Hirschhorn, 1999) suggest that there are likely to be challenges related to core assumptions, values and behavior when introducing and sustaining flexible and non-standardized approaches like Open Dialogue within a traditional, medically oriented mental health service.

Open Dialogue has often been positioned as an alternative approach to traditional mental health services, but without the research to understand more fully how the differing paradigms might be aligned effectively. This positioning of Open Dialogue, with minimal paradigmatic scrutiny, highlights the need for further exploration of the organizational and management challenges associated with the implementation of Open Dialogue. Further, we propose that Open Dialogue is more likely to flourish in a context where the style of leadership is adaptive, relational and responsive to changed conditions and ambiguity, where preference is given to dialogue and relationships over process (Heifetz, 1994; Lawrence, 2015; Scharmer, 2007; Schein, 2017), and where impact is measured in terms of experiences of personal recovery, rather than just symptom relief or management.

Brooks et al., (2011) commented on the lack of explanatory power of macro level theories for implementing innovative approaches in health care and the need to explore context, process and outcomes in more detail, when considering the effectiveness of implementing innovation, particularly in mental health settings. The current study responds to a lack of research on the organizational and leadership factors that influence the implementation of Open Dialogue approaches. We were interested in whether traditional models of change management were applicable to introducing Open Dialogue into a standard public mental health service. The study focused on two Australian healthcare organizations where there was sustained organizational and clinical implementation of Open Dialogue. The aim of the study was to identify and explore the organizational, management, leadership and cultural factors that contributed to a sustained implementation of Open Dialogue and to suggest prerequisite conditions for future implementations.

## Methods

The case study (Stake, 1995) was based on interpretations of transcripts of nine individual, semi-structured interviews with health care leaders and managers at two Australian sites, where Open Dialogue had gained sustained organizational and clinical traction over a five-year period.

### Study Contexts

Both research sites were child and youth mental health services characterized by an interest in working in a more collaborative, family-focused way and which had initiated Open Dialogue training of clinical staff members in early 2017.

Site A is the Child and Youth Mental Health Service (CYMHS) of a public health service in an outer metropolitan public health service that includes a rural area in New South Wales, Australia. The CYMHS comprise five teams, and Open Dialogue was implemented in two of these teams: the Early Psychosis Intervention Program (EPI), providing direct clinical services to a target population aged 12–25 years who were either experiencing or at risk of experiencing a first episode psychosis, and the Assertive Response Outreach Team (ARO) providing assertive and intensive therapeutic interventions for children and adolescents aged 5–18 years who were experiencing either acute behavioral or mental health problems. A significant part of a third, adult mental health team also participated in the training, but because it was situated very differently organizationally and had a different type and level of uptake, it was not included in this comparative analysis.

Site B is the child and youth mental health division of a large urban public health service in Victoria, Australia, comprising an Early Psychosis service and a tertiary CYMHS. The Early Psychosis service comprises five teams and sees young people aged 12–25 years from across a broad catchment area, while the CYMHS team sees young people from birth through to 25 years, along with their families, from across a smaller area of inner suburbs. While this CYMHS service has been established for many years, the Early Psychosis program was developed in 2013.

### Participants

Based on our prior knowledge of the settings, we used a snowball-sampling strategy (where enrolled participants assisted in identifying potential future participants) to recruit all leaders and managers that had had a significant involvement in the early implementation of Open Dialogue. In total, we invited ten leaders and managers to participate and nine responded and agreed to participate. We have no information regarding the reasons for the non-response. The participants included five from Site A and four from Site B. Among our participants, there were administrative and clinical leaders, operating at team, operational and executive leadership levels.

### Data-Generation

Interviews were conducted in April to May 2021 by E.L. who is an experienced qualitative research interviewer. They took place face-to-face (n = 7) or online (n = 2). All the interviews were audio-recorded. The interviews typically lasted approximately 60 min (range: 60–90 min.). Research assistants transcribed the recordings into written language, and the research team checked the accuracy of the transcriptions against the recordings. The interview guide was designed to facilitate and support the interpersonal relationship between respondent and interviewer, and to focus the interview on particular issues that were relevant to our research questions (Kvale & Brinkmann, 2014). The interview guide (See Table 1) was semi-structured to allow an exploration of the individual respondent’s perspective by following up on their concrete responses.

### Data Analysis

The thematic analysis began during the data collection phase, with the interviewer (E.L.) developing rudimentary memos and discussing these with another team member (N.B.). These initial discussions stimulated awareness and emphasis on certain topics in the subsequent interviews, for instance around ‘site narratives’ and leadership issues. When the interviews were fully transcribed, three researchers (N.B., L.H. and E.L.) independently read and coded the full dataset and used similarities and differences to discuss interpretations and emerging themes in shared documents and face-to-face meetings. This led to the collaborative identification of seven distinct themes that were reduced to three themes related to contextual leadership and management concerns. Through extensive memo writing, these themes were developed by adding analysis of richly contextualized data extracts. Memos were later reduced to fit the results section of the current paper.

### Ethics

The University of Sydney Human Research Ethics Committee approved the study at one site (reference #2020/155) and Alfred Hospital Ethics Committee at the other site (reference #702/20). Operating on the principle of “an arm’s length”, non-researchers mediated the first written information about the study. All participants gave their informed consent to participate based on written and oral information about the study. Responses were managed in full confidentiality. Given that important details about individual participants’ organizational positions were impossible to completely anonymize in the results section, we sent a draft to all participants and received their approval to publish the findings.

## Results

Findings are thematically organized around three headings: (1) “Initiation of a change process”, which is concerned with the situated origins of the Open Dialogue implementation processes, (2) “The context of leadership”, which is concerned with the organizational conditions for management and leadership, and (3) “Leadership in action”, which is concerned with the concrete change management practices used in the implementation of Open Dialogue.

**1. Initiation of a change process**: When asked about their initial interest in Open Dialogue, participants at both sites identified clients and carers as first-movers. Local communities had supported a number of presentations and seminars in Australia by internationally renowned Open Dialogue clinicians. This generated early interest amongst small groups of clinicians who shared journal articles and DVDs about Open Dialogue from Finland and attended international conferences. Interest was re-invigorated in 2017 when “The Open Dialogue Initiative” based at St. Vincent’s Private Hospital Sydney established more substantive training opportunities. At both sites, executive management had a strong tendency to support new research and development initiatives, such as Open Dialogue, as the CYMHS teams were regarded as well-functioning and relatively low-risk parts of the service. There was a high level of trust between executive management and clinicians, who were seen as exemplars in the way they observed required risk protocols and still maintained a high level of empathy and responsiveness to client needs. This ability to operate ‘under the executive’s radar’ by managing medical and organizational risk points was expressed clearly by a member of Site A senior management group:

*We were managing waitlists, we weren’t coming up as repeatedly having people in emergency, so there was no reason to put the spotlight on CYMHS. I think within whatever we were doing we were still meeting the clinical risk management expectations of the organization. (Tyler)*

Furthermore, the promise of Open Dialogue was well aligned with other family-oriented initiatives in the service and thus became part of larger local service initiatives.

The establishment of Open Dialogue at Site A was largely based on a bottom-up implementation, supported by a stable and relatively large group of committed and family therapy (systemically) trained clinicians. Three teams at Site A joined a training and research program. The program included five in-house pre-training sessions, a one-week training program by Scandinavian Open Dialogue trainers, and in-house post-training supervision as well as research on the acceptability of the training for participants. Participation was voluntary for clinicians, but most team members took part in the training. The Open Dialogue Initiative supported the training, so costs beyond ‘back-filling’ of staff were minimized for the service. The research component was crucial for the program’s legitimacy in the organization as senior management had to formally ‘sign off’ on the site’s participation in research. However, this formal approval also lent legitimacy to the wider, ongoing clinical implementation processes. Clinicians at Site A were largely allowed to work with a range of therapeutic models, so clinicians who did not embrace Open Dialogue could legitimately work with an emphasis on other approaches.

The establishment of Open Dialogue at Site B was predominantly driven by a top-down approach organized by a tightly knit senior management group with a keen interest in working collaboratively with clients and carers and who had an awareness of some shortcomings of conventional medical models. Members of the senior management group at Site B had become enthused after participating in training sessions offered by the Open Dialogue Initiative. A unique, large short-term funding opportunity allowed the group to establish a training course that included five partly in-house pre-training sessions and one-week training by Australian Open Dialogue trainers linked to the Open Dialogue Initiative. Executive management approved the training, which was strongly encouraged for members of the five teams, and the ongoing managerial commitment combined with the externally-driven training program added to legitimizing the Open Dialogue implementation.

**2. The context of leadership**: Open Dialogue, as it has developed as a practice, has two core components – the dialogical approach (requiring practice change) and the service delivery approach (requiring organizational change) (Olson et al., 2014). Implementing the change towards Open Dialogue in mental health services therefore required services to address leadership and power relations across two dimensions: the clinical (medical) and the organizational (administrative-managerial). While these two dimensions of services operate in different realms, there is overlap in clinical decision-making and risk management processes. While the implementation of Open Dialogue required leadership in both dimensions, the internal and external constraints and drivers for each were very different. The two sites approached the change process in different ways, demonstrating differences in their approaches to clinical and organizational leadership.

The bottom up approach at Site A was largely driven by clinical staff who were inspired to improve practice and enhance outcomes for young people and their families. In particular, the endorsement of Open Dialogue by psychiatrists on the clinical teams was a critical component, which drove implementation in practice and influenced decision making and support at the organizational leadership level. The top down approach at Site B was driven by a consortium of clinical and organizational leaders who were inspired to create a values-driven service, which met the needs of young people and families as well as conforming to very restrictive constraints in the model of care, some of which were highly antithetical to Open Dialogue. For instance, at both sites, pressures to conform to externally imposed requirements relating to issues such as access to formalized and accredited training, resource and funding restraints and reporting requirements limited the extent to which structural change could be internally enacted. Illustrating this, Tony, a middle manager at site B, stated.

*Essentially it’s a medical model that’s imposed on us. There is a [external] requirement to give an evidence-based treatment and for people to have a medical review within a particular time frame. And we’re marked on doing “symptom checklists” things like that. It undermines, I think, dialogic practice.*

There were also internal constraints to practice change, particularly when differences of clinical opinion emerged within teams. The power of psychiatrists within mental health care emerged as an issue if those personnel were not onboard with a shift towards Open Dialogue. This may particularly have been exacerbated because of the requirement for clinicians to loosen their grip on power in the Open Dialogue approach. This could equally or alternatively be seen as increasing risk, where risk in mental health is traditionally held by the psychiatrists, thus vesting a formal authority in the medical role. A respondent, Kim from Site B, noted: *There’s a lot of Psychiatrists in powerful positions who are anti it [Open Dialogue] because it does threaten the power of psychiatrists.*

An area of the administrative organization that had a significant impact on both sites was funding and resourcing arrangements. Site A was advantaged by having continuous public funding at the service level and a management team, who were able to secure funding streams for ongoing training and backfilling positions, when practitioners were on leave or in training. In Site B, the funding structure was complex, as it was based on a three-year cycle and attached to specific goals and performance indicators that did not clearly align with Open Dialogue practices. This required constant effort and adaptation by the leadership of the Open Dialogue program in two important areas: (1) the recruitment, training and enculturation of new practitioners every two to three years and (2) the renegotiation of funding, and the ongoing management of the relationship with the funding body. The outcome of this inconsistent, time limited resourcing was felt at the practitioner level in terms of limited and inconsistent opportunities to build depth of capability, and at the management level as a continuous round of negotiations, recruitment and induction of new staff.

**3. Leadership in action**: We found differences in the way leaders introduced, managed and sustained the changes required to implement Open Dialogue. These variations were often a function of the level of leadership and type of role, that is clinical versus organizational leadership. The most critical role both clinical and organizational leaders described themselves as enacting was that of managing or removing barriers for practitioners, so that they were free to explore and develop as Open Dialogue practitioners.

At Site A, organizational and clinical leaders at each level (from team manager to executive levels) worked to create resourcing and governance structures that brought stability and depth of capability to the Open Dialogue program. Creative leadership actions such as formalizing training programs and re-designing funding processes so that ongoing training programs were embedded in the program were undertaken to enable these changes to occur. Leaders were proactive in shepherding mechanisms such as the Open Dialogue research project and ongoing training program through the health bureaucracy, which then created legitimacy for Open Dialogue’s transition from an idea to a formal program across multiple teams.

While the Executive leaders at Site A were committed and supportive of Open Dialogue, they left the work of defining vision and aspirations to the clinical or team manager levels, which is not surprising given that they were not clinical in their orientation and hence had less personal passion and commitment to Open Dialogue. They viewed Open Dialogue as “making good business sense” and were eager to support a clinical team that had a positive track record.

An executive level leader at Site B, Kim, also had a clinical role, and played a similar organization building role, through identifying an important funding opportunity and managing its passage through the complexity of state and federal bodies, which then enabled more effective staffing, including the development of an ongoing Open Dialogue training program. Reflecting on leading and supporting the Open Dialogue implementation process, Kim stated: “*Some of these people need help to implement [Open Dialogue]. And it’s mainly about removing barriers and dealing with politics. So my job is really that”.* In addition, Kim described playing a visionary, and to an extent, transformational leadership role (Bass & Avolio, 1994) in defining the scope and aspiration of the Open Dialogue program for the entire site, while continuing to manage resistance at both clinical and organizational management levels.

Another critical role that leaders at both sites described was in influencing significant actors both within their health system and externally, to create legitimacy for the Open Dialogue program and ensure consistent funding. This management of stakeholders was particularly critical in Site B, and required cultivating an ongoing discussion and strong relationship with an important and highly visible, influential external stakeholder to ensure that there was trust and confidence in Open Dialogue’s capacity to respond both to the funding bodies’ requirements and the site’s internal benchmarks.

At the practitioner level, particularly for Site B, there was a strongly felt dissonance between the formal reporting requirements, the statutory accountabilities of psychiatrists around clinical risk and the ethos and practice of Open Dialogue. This required a leadership response that was attuned to this complexity and worked to both explore and face the tensions at the same time as managing the interface with the health system, particularly in relation to risk and reporting requirements. At both sites, leaders were highly skilled at performing this dual role of managing down into the organization, and managing above. Speaking about leadership, Ash, a Team manager at Site A stated:

*I draw on a lot of different theories to inform how I manage, and Open Dialogue has been a significant one. A lot of management is of clinical work with staff, you know dealing with people who are coming with issues and need to be, so I think that idea around creating a space where people feel safe to speak about the issues that they have, making sure that they feel heard and responded to is a good way to do management.*

Leaders at all levels reflected on the way in which learning from Open Dialogue, particularly the dialogic skills and mindset acquired with experience, influenced their own leadership style and management of workplace conflict and decision-making. This personal change began to influence the culture of both services through practices such as open communication and conflict management.

## Discussion

Reflecting on the implementation of Open Dialogue practices in conventional mental health service structures, the participants described an unfinished and messy process of gradual clinical and organizational change rather than a streamlined change management process, with a beginning and end. We interpreted their leadership styles as highly flexible as they responded to community pressures and complex, often contradictory, organizational factors. Most notably, leaders facilitated the gradual development of clinical and organizational legitimacy for the non-standardized Open Dialogue approach by holding the anxiety and frustration of practitioners and parts of the administration and by continually removing organizational obstacles (Heifetz, 1994; Heifetz & Laurie, 2001). We believe that our analysis of this process is a comprehensive answer to Brooks et al.’s (2011) call for more situated analyses to explain organizational change processes.

An important feature of our analysis is the observation of cultural change at both sites. Understanding organizational culture usually begins with an exploration of assumptions and values associated with a group (Heifetz, 1994; Lawrence, 2015; Schein, 2015). The presence of similar values, such as trust in and between staff, the cultivation of respectful relationships and a commitment to ongoing learning and development as an Open Dialogue practitioner, were consistently commented on by interviewees. These values were framed as being essential elements of the organizational culture that underpinned both programs. Drawing on these core values and practices offered both leaders and practitioners a way of managing interpersonal, professional and practice tensions.

Leaders demonstrated their critical role in facilitating change at this deeper level when they allowed these tensions to surface and be aired, which sometimes led to resolution and at other times, acceptance of differences (Marshak & Grant, 2008). On the surface, values held the teams together but there remained tensions between those that were keen Open Dialogue practitioners versus those that were not as committed or held a different perspective on treatment. Over time, participants commented that the values had become embedded in both services, creating a consistency and coherence of culture at both the clinical practice and organizational levels, especially within the groups that actively embraced Open Dialogue (Carroll & Quijada, 2004).

The question the researchers asked themselves at a number of points during this project was whether this was a story about Open Dialogue implementation or was it about implementing an innovation that involved cultural change in a complex context. We lean towards the latter, as our findings indicate the value of conceptualizing the introduction of a program such as Open Dialogue as a nonlinear and adaptive process in a complex, open system. The alternative is to calibrate our findings against the principles of traditional “change management” models (Kotter, 2012; Weisbord, 2012). These traditional models offer both process and descriptive approaches to organizational change but essentially draw on similar themes: the importance of a sense of urgency and commitment at the top of the organization, the power of vision and goals to define and drive the direction of change, a step by step process that unfolds in a predictable, linear manner, supported by clear leadership communication and training focused on roles and behavior.

Some of the elements described in these models are consistent with Brookes et al.’s (2011) model for innovation in health care and our findings do provide insights that align with a more traditional, process oriented approach to change management. Factors such as the value of committed leadership, clear communication, ongoing training and learning, awareness and management of resistance to change were present across both sites. We did, however, find evidence of a different style of leadership to the top down or transformative leadership approach often described in the change management literature (Akinbode & Al Shuhumi, 2018; Kotter, 2012; Kouzes & Posner, 2017).

Health systems have been identified in the literature as complex adaptive systems (Ellis, 2013; Lane et al., 2021; Sturmberg et al., 2012) and the leadership proposed as most effective in these contexts is ”adaptive leadership” (Heifetz & Linsky, 2017). At the heart of this style of leadership is an adaptive challenge to be addressed. Heifetz & Linsky (2017) describe this as a systemic change that requires a shift in assumptions, values and behavior for which there is no clear precedent or known process, that is, an adaptive challenge is not a technical problem to be solved through a known and tested procedure. The implementation of Open Dialogue in a traditional mental health care setting can be characterized as an adaptive challenge because it not only requires changes to the model of care, but additionally, significant adjustments in terms of professional skills, assumptions about mental health, values and behavior.

The style of leadership that emerged from participant accounts is reflective of the characteristics described in adaptive leaders working in complex contexts (Heifetz, 1994; Lane et al., 2021; Snowden & Boone, 2007). These include the capacity to sense what is happening beneath the surface of teams, to be responsive in the moment, to change course and be flexible where necessary. We consistently heard stories of leaders at every level protecting the voices of practitioners, allowing the tensions between differing voices to exist and emerge, but at the same time facilitating the relational and reflective work required for people to safely explore the challenges to clinical assumptions, values and practices that emerged from Open Dialogue (Heifetz & Laurie, 2001; Heifetz & Linsky, 2017). While there are elements in common with transformational leadership, adaptive leadership calls for a more nuanced approach to change and being present to the unconscious dynamics operating in groups (Heifetz, 1994; Scharmer, 2007).

A critical feature of adaptive leaders is their capacity to create a “holding environment”. This is essentially the skillful balance between creating symbolic and tangible anchors to engender a sense of psychological safety, and placing pressure on people at every level in the organization to do the deeper reflective work described above (Heifetz & Laurie, 2001; Heifetz & Linsky, 2017). The concept of a holding environment is intertwined with the notion of organizational context and culture (Van Buskirk & McGrath, 1999) and has been applied both to the process of individual growth and change, as well as organizational change (Armstrong, 2005; Heifetz, 1994; Kegan, 1994). Leaders who are able to create an effective holding environment limit the anxiety in the system (Krantz, 1998), which enables people and the social system in which they work to experiment, learn, change and adapt so that there is continuity and sustainability.

The leadership style enacted by study participants seemed to work effectively in navigating the shifting elements of the administrative and clinical systems, the challenges of supporting clients in crisis, and the tensions related to different expertise and preferred treatment modalities among practitioners. It also contributed to the co-creation of an organizational culture that bridged the values underpinning Open Dialogue and also those of the health care facility to create a shared narrative (Marshak & Grant, 2008). These values were described by participants as trust, collaboration, openness and transparency, curiosity and ongoing learning. While practitioners sometimes expressed disappointment that the Open Dialogue implementation was not as advanced or extensive as they had imagined it would be when they began the journey, from a change perspective, both sites have sustained an Open Dialogue program over at least 5 years and there is evidence of further growth and development to come.

### Limitations

Our focus on leadership and management factors in Open Dialogue implementation meant that the study population was of a limited size. However, the nine participants can be regarded as ‘key informants’ with a unique first-hand insight into the study subject. We are cautiously aware that there may be gaps between the perspectives of the study participants and other stakeholders in the organizational change processes. Future research could include explorations of alternative or counter-narratives, which could add to a more holistic understanding of the complexities of sustained organizational and cultural change. Finally, results were based on highly collaborative research processes that both inspired creative interpretations and - because of an inherent need for consensus - added to creating balanced readings of the dataset.

## Conclusions

In summarizing their findings and model of innovation in health care design, Brooks et al., (2011) reflect similar sentiments to those observed in this research across the two sites: *“…. because predictions are very difficult to make in open systems, innovations will only be united by leaders and managers developing a self-conscious and determined approach to organisational learning and the need to nurture adaptive organisations”*.

The two sites we explored were both graced by committed and intuitively skillful, adaptive leaders who championed change and managed organizational stakeholders effectively, both key elements in Brooks et al.’s (2011) conclusions about successful innovations in health care. The conditions in terms of funding possibilities and organizational readiness for the implementation of a new model of care were also favorable. Readiness is often described in the change literature as a felt need for change (Kotter, 2012). Additionally, the two sites were ‘change ready’ in that practitioners were seen as highly credible and skilled at managing clinical risk, hence organizational leadership trusted them and their leaders, and were supportive of innovation and change, rather than resistant. The processes for training and ongoing practice development were identified early in the process as important, and both sites were able to commit resources and time to that endeavor, which added to skill levels, confidence and also cultural cohesion. This cultural cohesion was both a function of the values and outlook of Open Dialogue oriented clinicians and the way in which leaders were able to create rich “holding environments” that fostered dialogue, supported continuity and change, and allowed meaningful sense making to occur (Grant et al., 2005; Weick et al., 2005).

Public health systems are complex and notoriously hard to change. In the introduction, we noted the recent characterization of the system as “broken” and in need of improvement. There are always calls for more client centered care, more involvement of the broader community as a resource. Health systems, however, are large bureaucracies, with an evolved business model and power structure that drives a momentum that is not always in line with improved patient care and innovation. The concerns and accountabilities of administration and clinicians are not the same and hence innovation at the service level always involves stakeholders with different requirements. This paper demonstrates that innovation and significant change in service delivery is possible but there are constraints and one of these is where the model of leadership and change do not fit the complexity of the situation.

**Table 1:** The interview guide

1.	What is your experience of working with the Open Dialogue approach as it has been implemented in your organization?
2.	How was Open Dialogue introduced to your organization? Were you part of the original implementation team? If not, how long have you been involved with the Open Dialogue project?
3.	Can you tell me about your experiences from the perspective of your organizational role?
4.	What aspects of Open Dialogue have you found easiest to implement in your organization?
5.	Are there structural, cultural or leadership factors that have worked to enable the implementation?
6.	Have you discovered any challenges or barriers, from a structural, cultural or leadership perspective?
7.	How have you sought to manage those challenges? Have those strategies been effective?
8.	How has your service changed since the introduction of Open Dialogue?
9.	Have there been personal or organizational adjustments that you or your colleagues have had to make?
10.	How has the introduction of Open Dialogue impacted your approach, engagement or role within your organization and way of working?
11.	Looking back, are there organizational, leadership or people aspects that you have learnt might work as either enablers or barriers to future implementations?
